# Preventive Impella^®^ Support in High-Risk Patients Undergoing Cardiac Surgery

**DOI:** 10.3390/jcm11185404

**Published:** 2022-09-14

**Authors:** Kálmán Benke, Edina Korça, Anniek Boltjes, Roland Stengl, Britt Hofmann, Meradjoddin Matin, Katharina Krohe, Yuliana Yakobus, Jens Michaelsen, Levan Khizaneishvili, Gábor Szabó, Gábor Veres

**Affiliations:** 1 Department of Cardiac Surgery, University Hospital Halle (Saale), Martin-Luther University Halle-Wittenberg, 06120 Halle, Germany; 2 Heart and Vascular Center, Semmelweis University, 1122 Budapest, Hungary

**Keywords:** Impella, ECLS, CABG, postcardiotomy cardiogenic shock

## Abstract

Background: Patients with severely reduced LV-EF ≤ 30% undergoing CABG have a high risk for postoperative cardiogenic shock. The optimal timing of an adequate hemodynamic support has an impact on short- and midterm mortality after CABG. This study aimed to assess the prophylactic use of the Impella pump in high-risk patients undergoing elective cardiac surgery. Methods: In this single-center retrospective study, 14 patients with LV-EF (≤30%) undergoing cardiac surgery received a prophylactic, perioperative Impella (5.0, 5.5) support between 2020 and 2022. Results: The mean age at surgery was 64.2 ± 2.6 years, the mean preoperative LV-EF was 20.7% ± 1.56%. The duration of Impella support was 4 (3–7.8) days and the 30-day survival rate was 92.85%. Acute renal failure occurred in four patients who were dialyzed on average for 1.2 ± 0.7 days. Mechanical ventilation was needed for 1.75 (0.9–2.7) days. Time to inotrope/vasopressor independence was 2 (0.97–7.25) days with a highest lactate level (24 h postoperatively) of 3.8 ± 0.6 mmol/l. Postoperative LV-EF showed a significant improvement when compared to preoperative LV-EF (29.1% ± 2.6% vs. 20.7% ± 1.56% (*p* = 0.022)). Conclusion: The prophylactic Impella application seems to be a safe approach to improve the outcomes of this patient population.

## 1. Introduction

Postcardiotomy cardiogenic shock (PCS) is a serious complication affecting approximately 0.2–6% of patients undergoing cardiac surgery and it is defined as a low cardiac output syndrome with hypoperfusion of tissues and evidence of end-organ dysfunction in the presence of adequate preload [1]. PCS refractory to pharmacologic measures, intravascular volume loading and intra-aortic balloon pump support develops in about 0.5–1.5% of cardiac surgical patients and it necessitates the use of extracorporeal life support (ECLS) [1,2]. The mortality rate of PCS is around 50–80% [1], which underlines the significance of preoperative identification of patients with a high risk for PCS and the application of preventive measures in order to improve their postoperative outcomes. Patients with severely reduced left ventricular ejection fraction (LV-EF) undergoing coronary artery bypass grafting (CABG) are at an increased risk for PCS and their mortality rate after the operation is about 6.5% [3].

Impella 5.0 and 5.5 are transvalvular microaxial pumps that deliver up to 5 and 5.5 mL/min blood, respectively, from the left ventricle to the aorta, thereby reducing the workload of the heart and improving cardiac output [4]. Their application for the treatment of PCS has been proven to be safe and beneficial [5]. This finding, together with the principle of the working mechanism and minimally invasive implantation technique, could make the use of the Impella device a promising option to prevent the development of PCS after cardiac surgery. While various studies are available on the prophylactic use of Impella in high-risk patients undergoing percutaneous coronary intervention (PCI), the application of Impella as a preventive therapeutic option for PCS in high-risk patients undergoing cardiac surgery has scarcely been reported [6].

Thus, the aim of this study was to analyze the outcome of patients with severely decreased left ventricle function (LV-EF ≤ 30%) undergoing elective cardiac surgery with the application of prophylactic Impella support. 

## 2. Materials and Methods

In this single-center retrospective study, 14 patients with severely reduced LV-EF (≤30%) undergoing cardiac surgery (CABG; aortic valve replacement (AVR); CABG with AVR or CABG with mitral valve reconstruction (MVR)) received a prophylactic Impella support between 2020 and 2022. Other inclusion criteria besides a highly reduced LV-EF (≤30%) were elective cardiac surgery, comorbidities (chronic kidney disease, diabetes mellitus, COPD, peripheral artery disease, atrial fibrillation, arterial hypertension, or dyslipidemia) and chronic heart failure. Exclusion criteria were the need of urgent operation, preoperative need of mechanical support, preoperative need of catecholamines and decompensated heart failure. Four patients were preconditioned with a calcium sensitizer (Levosimendan), one of them 10 days prior to the operation. The patients gave their written consent to this approach of surgery with Impella implantation.

### 2.1. Surgical Technique

During the main operation, Impella devices were implanted under general anesthesia in the same manner as previously described [7]. The right axillary artery was accessed and a 10 mm vascular graft was anastomosed to the artery with 6/0 prolene running suture before surgery. After securing the correct position of the pump with transesophageal echocardiography and fluoroscopy guidance, the vascular graft was shortened to the skin level and the system was appropriately secured. The incision was closed in anatomical layers. Intravenous heparin was provided immediately before cross-clamping of the axillary artery to perform the anastomosis (target-activated clotting time (ACT): 240 s). 

### 2.2. Weaning Criteria

After being stabilized on Impella support, the patients were treated with (Levosimendan) over 24 h prior to the weaning process. 

The patients were monitored with ICU monitoring (arterial line, central venous line, and PICCO), and the unloading was assessed echocardiographically. The right ventricular function was assessed echocardiographically and based on the surrogate clinical parameters (ScvO_2_, CVP). Impella 5.0 was used for support in all but one patient who received an Impella 5.5 device.

Weaning was performed according to the protocol proposed by Balthazar et al. with slight modifications [8]. Before starting with the weaning process, the patients had to be extubated, had to have normal lactate levels, needed only low doses or did not need vasopressor or inotrope therapy. When this was achieved, the Impella support was reduced gradually down to 2.5 L support for 2 h. Echocardiographic examination was performed before explantation to assess LV-EF. If after this time the patient’s hemodynamics remained stable, the Impella was explanted at the patient’s bedside.

### 2.3. Statistical Analysis

Patient characteristics, pre- and postoperative data, comorbidities, complications and 30-day mortality were analyzed. Continuous variables are presented as mean ± standard error of the mean (SEM) or median with interquartile range (IQR), categorical variables are presented as numbers and percentages. Normality was checked using a Shapiro–Wilk test and a paired sample t-test was used to compare the pre- and postoperative LV-EF values. A *p* value < 0.05 was considered statistically significant. 

## 3. Results

### 3.1. Baseline Characteristics, Preoperative and Operative Data

Preoperative patient characteristics and operative data are summarized in Table 1. A total of 14 patients with a mean LV-EF of 20.7% ± 1.56% who underwent cardiac surgery received a prophylactic Impella support. Of those, 10 (71.4%) underwent isolated CABG (one patient off-pump CABG), 2 (14.3%) AVR and 2 (14.3%) CABG and valvular surgery. All patients had multiple comorbidities. 

### 3.2. Impella Support

Thirteen patients (92.85%) survived to Impella explantation, and twelve of the Impella devices were removed in the ICU. The median duration of Impella support was 4 (3–7.8) days. Device related complications occurred in two patients. In one of these cases the removal of the Impella could not be carried out in the ICU, it had to be removed in the operating room under fluoroscopic guidance. In the other case the Impella was dislocated and satisfactory reposition could not be achieved. That patient suffered cardiogenic shock and received an urgent ECLS. No Impella device malfunction was reported. The Impella related data is demonstrated in Table 2.

### 3.3. Postoperative Outcomes

Postoperative data are shown in Table 3. Mean ICU stay was 11.3 ± 1.6 days, while mean hospital stay was 19.5 ± 2 days. Three patients received antibiotics during their hospitalization due to pneumonia. Acute renal failure occurred in 4 patients who were dialyzed in average for 1.2 ± 0.7 days. The duration of mechanical ventilation was 1.75 (0.9–2.7) days. Time to inotrope/vasopressor independence was 2 (0.97–7.25) days. 

The most recently determined LV-EF was 29.1% ± 2.6%. The 30-day survival rate was 92.85%. The postoperative LV-EF showed a significant improvement compared to the preoperative values (20.7% ± 1.56% and 29.1% ± 2.6%, respectively, *p* = 0.022). 

## 4. Discussion

In the current retrospective study, we present our experience of the prophylactic use of the Impella device (5.0, 5.5) in high-risk patients undergoing elective cardiac surgery. Apart from one case, all patients could be successfully weaned from the device, no Impella malfunction was reported, and the 30-day survival was over 90%, thereby demonstrating the prophylactic Impella support to be a potentially safe and beneficial approach in the management of high-risk cardiac surgical patients. The findings may add valuable information to the existing evidence on the safety and efficacy of this preventive approach, thereby contributing to the necessitation of further research. 

Patients with severely reduced LV-EF and comorbidities undergoing cardiac surgery are at increased risk for PCS. Once in need of ECLS due to PCS, their mortality rate increases to 50–80% [1]. Associated complications such as a major neurological event, renal failure requiring hemofiltration, lower limb ischemia and reoperation due to mediastinal bleeding are common [9]. 

Preventive Impella implantation in patients with poor LV function can offer a hemodynamic support of up to 5.5 L/min, thereby reducing the risk of PCS and its complications. Additionally, LV unloading due to Impella might optimize conditions for myocardial recovery and partially be responsible for the significant improvement in postoperative LV-EF. Impella is currently applied in cases of high-risk PCI and cardiogenic shock [4]. David et al. assessed the outcomes of 29 patients receiving the Impella 5.0 or Impella Left Direct (LD) device for the treatment of PCS. They reported a 58.6% 30-day and 51.7% 1-year survival, thereby demonstrating a benefit of this therapeutic approach [5], compared to the 50–80% mortality rate normally associated with PCS. So far, the application of Impella for the prevention of PCS has not been widely investigated. However, as shown by Iannaccone et al., the timing of Impella placement is of great importance in the improvement in patient outcome. Their analysis suggests that Impella implantation prior to PCI in acute myocardial infarction complicated by cardiogenic shock may have a positive impact on short- and midterm mortality with similar safety outcomes compared with post-PCI [10]. 

The preventive use of Impella support in high-risk cardiac surgical patients was demonstrated in a few case reports and case series, showing that mechanical support with this device could be a safe and promising therapeutic approach in this patient cohort [11,12,13,14]. 

One study reported the implantation of Impella LD (*n* = 10) and 5.0 (*n* = 3) in 13 high-risk patients undergoing CABG. Eight of these patients presented with acute coronary syndrome and four patients were admitted with acute decompensated heart failure. More than 60% of the patients were extubated within 48 h and out of bed within 72 h. The average duration of Impella support was 5.7 days and no postoperative deaths were reported. The Impella devices were implanted either via the right axillary artery (Impella 5.0) or via the ascending aorta (Impella LD) and they were explanted in the operating room [15]. However, we prefer implantation of the Impella 5.0 and 5.5 via the right subclavian artery due to the possibility of early weaning as well as mobilization of the patient with indwelling Impella and the ease with which it can be removed. Initially, the devices were explanted in the operating room; nowadays, we perform this in the ICU without sedation, which could be beneficial because of fast extubation and mobilization.

Currently used approaches to prevent PCS in patients with severely reduced LV-EF undergoing cardiac surgery are the preoperative administration of levosimendan or hemodynamic support via IABP. However, studies could not show definitive benefits of these approaches on patients undergoing different types of cardiac surgery. Studies showed only some benefit from preoperative use of levosimendan in patients undergoing isolated CABG [16,17,18]. While a meta-analysis of randomized trials came to the conclusion that prophylactic IABP use may reduce short-term mortality and major adverse cardiac and cerebrovascular events in high-risk patients with a mean LV-EF of 35% undergoing elective or urgent CABG [19], another study was unable to determine survival advantages of using prophylactic IABP in high-risk patients undergoing CABG [20].

ECLS is the current standard approach to treat PCS after cardiac surgery. However, this treatment has only very limited success with a high mortality and complication rate.

To the best of our knowledge, the prophylactic use of Impella in cardiac surgery has only been investigated in patients undergoing CABG. The current work also involves results in case of valve operations, and thus broadens the spectrum of the possible applications of the presented device. 

## 5. Limitations

The current study has some limitations, of which the most important one is the small sample size. However, mostly case reports and studies with a similar number of patients have been reported on this topic, meaning that our findings could have a relevant contribution to the field. Further limitations are the retrospective design and the lack of a control group. Despite these, our study provides important information on prophylactic Impella use in high-risk cardiac surgical patients, which could provide a base for further studies.

## 6. Conclusions

The presented findings extend the currently available experience with the prophylactic use of the Impella device in high-risk cardiac surgical patients. The application of the Impella in this setting seems to offer a safe method to improve the outcomes of this patient population; however, prospective multi-center studies are warranted to further examine the feasibility and benefits of this prophylactic approach.

## Figures and Tables

**Table 1:** Preoperative patient characteristics and operative data.

	Total (*n* = 14)	CABG (*n* = 10)	Aortic Valve Surgery (*n* = 2)	CABG and Valvular Surgery (*n* = 2)
**Preoperative characteristics**				
Male	13 (92.86%)	9 (90%)	2 (100%)	2 (100%)
Age (years)	64.2 ± 2.6	64.6 ± 3.06	58.5 ± 1.5	68 ± 12
BMI (kg/m²)	28 ± 1.4	29.2 ± 1.7	25 ± 4.04	25 ± 2.6
Body surface area (m^2^)	1.98 ± 0.07	2.0 ± 0.09	1.87 ± 0.15	1.84 ± 0.1
LV-EF (%)	20.7 ± 1.56	21.9 ± 1.28	15.5 ± 3.5	20 ± 10
Smoking	10 (71.4%)	8 (80%)	2 (100%)	0 (0%)
Dyslipidemia	2 (14.3%)	2 (20%)	0 (0%)	0 (0%)
Arterial hypertension	11 (78.6%)	10 (80%)	1 (50%)	2 (100%)
Peripheral artery disease	4 (28.6%)	2 (20%)	2 (100%)	0 (0%)
Chronic kidney disease	3 (21.4%)	2 (20%)	0 (0%)	1 (50%)
COPD	6 (42.9%)	3 (30%)	2 (100%)	1 (50%)
Atrial fibrillation	6 (42.9%)	4 (40%)	1 (50%)	1 (50%)
Chronic heart failure	11 (78.6%)	8 (80%)	1 (50%)	2 (100%)
Ischemic cardiomyopathy	6 (42.9%)	4 (40%)	1 (50%)	1 (50%)
Dilated cardiomyopathy	4 (28.6%)	3 (30%)	1 (50%)	0 (0%)
Prior myocardial infarction	5 (37.7%)	4 (40%)	0 (0%)	1 (50%)
Serum creatinine (µmol/l)	88.7 ± 5.7	87.2 ± 6.7	78.5 ± 9.5	106.5 ± 19.5
Bilirubin (µmol/l)	12.85 (7.3–15.98)	10.95 (7.3–19.35)	9.3 (7.15–11.45)	15.95 (15.925–15.975)
Lactate (mmol/l)	1.18 ± 0.11	1 ± 0.12	1.8 ± 0	1.35 ± 0.25
GFR (ml/min)	88.5 (76.5–90)	87.5 (76.5–90)	90 (90–90)	66 (55.5–76.5)
**Operative data**				
Heart lung machine time (min)	152 ± 17.1	140.5 ± 20.8	200.5 ± 62.5	161 ± 15
Aortic cross-clamp time (min)	51.5 ± 6.2	44.7 ± 6.1	60.5 ± 17.5	76.5 ± 24.5

Categorical variables are expressed as *n* (%), and continuous variables are expressed as mean ± standard error of the Mean and median with interquartile range. SEM, standard error of the Mean; CABG, coronary artery bypass grafting; BMI, body mass index; BSA, body surface area; LV-EF, left ventricular ejection fraction; COPD, chronic obstructive pulmonary disease; GFR, glomerular filtration rate according to CKD-EPI; Valvular surgery include: Aortic valve replacement and mitral valve reconstruction

**Table 2:** Impella related data.

	Total (*n* = 14)	CABG (*n* = 10)	Aortic Valve Surgery (*n* = 2)	CABG and Valvular Surgery (*n* = 2)
Duration of Impella support (days)	4 (3–7.8)	4 (3–6.5)	11.5 (7.75–15.25)	5.5 (4.25–6.75)
Survival to Impella explant	13 (92.85%)	10 (100%)	2 (100%)	1 (50%)
Device related complications	2 (14.3%)	2(20%)	0 (0%)	0 (0%)
Impella malfunction	0 (0%)	0 (0%)	0 (0%)	0 (0%)

Categorical variables are expressed as *n* (%), and continuous variables are expressed as mean ± standard error of the Mean and median with interquartile range.

**Table 3:** Postoperative patient characteristics.

	Total (*n* = 14)	CABG (*n* = 10)	Aortic Valve Surgery (*n* = 2)	CABG and Valvular Surgery (*n* = 2)
Mechanical ventilation in the ICU (days)	1.75 (0.9–2.7)	2.5 (0.8–2.7)	1.71 (1.69–1.73)	2.1 (1.7–2.5)
Acute renal failure	4 (28.6%)	3 (30%)	0 (0%)	1 (50%)
Duration of renal replacement therapy (days)	0 (0–1.5)	0 (0–1.5)	0 (0–0)	1 (0.5–1.5)
ICU stay (days)	11.3 ± 1.6	11.6 ± 1.8	12 ± 7	9 ± 6
Hospital stay (days)	19.5 ± 2	20.3 ± 1.9	16.5 ± 3.5	18.5 ± 13.5
30-day survival rate	13 (92.85%)	10 (100%)	2 (100%)	1 (50%)
Pneumonia	3 (21.4%)	2 (20%)	0 (0%)	1 (50%)
Time to lactate normalization (h)	15.4 ± 2.8	16.3 ± 3.5	16.1 ± 2.1	4.8 ± 0
Time to inotrope/vasopressor independence (days)	2 (0.97–7.25)	2 (0.6–5.8)	1.7 (1.7–1.7)	11.7 (11.7–11.7)
Most recent LVEF (%)	29.1 ± 2.6	32.7 ± 1.6	28 ± 3	12 ± 12
Lactate (24 h postoperatively highest) (mmol/l)	3.8 ± 0.6	3.2 ± 0.4	4.3 ± 1.2	6.5 ± 3.7
Transfusion of erythrocyte concentrates	11.5 (8–16.8)	11.5 (8.5–16)	11.5 (7.8–15.3)	15.5 (11.8–19.3)
Transfusion of fresh frozen plasma	4.5 (4–13.3)	4.5 (4–11)	13.5 (8.8–18.3)	13 (8.5–17.5)
Transfusion of thrombocyte concentrates	2.8 ± 0.6	2.8 ± 0.9	2.5 ± 0.5	3 ± 1
Postoperative bleeding (<24 h)	1 (7.15%)	1 (10%)	0 (0%)	0 (0%)

Categorical variables are expressed as *n* (%), and continuous variables are expressed as mean ± standard error of the mean and median with interquartile range.
